# Unfixed Movement Route Model, Non-Overcrowding and Social Distancing Reduce the Spread of COVID-19 in Sporting Facilities

**DOI:** 10.3390/ijerph18158212

**Published:** 2021-08-03

**Authors:** Bote Qi, Jingwang Tan, Qingwen Zhang, Meng Cao, Xingxiong Wang, Yu Zou

**Affiliations:** 1 Department of Sport and Exercise Science, College of Education, Zhejiang University, 886 Yuhangtang Road, Hangzhou 310058, China; qibote@zju.edu.cn (B.Q.); preder@yeah.net (J.T.); 2 College of Physical Education and Training, Shanghai University of Sport, 399 Chang Hai Road, Shanghai 200438, China; zqw@sus.edu.cn; 3 Institute of Physical Education, Normal College, Shenzhen University, 3688 Nan Hai Road, Shenzhen 518061, China; caomengsus@163.com; 4 College of Management and Economics, Tianjin University, 92 Wei Jin Road, Tianjin 300072, China; wxx98@tju.edu.cn

**Keywords:** COVID-19, sports facilities, agent-based modeling, NetLogo simulation

## Abstract

Localized outbreaks of COVID-19 have been reported in sporting facilities. This study used the Agent-based Modeling (ABM) method to analyze the transmission rate of COVID-19 in different sporting models, sporting spaces per capita, and situations of gathering, which contributes to understanding how COVID-19 transmits in sports facilities. The simulation results show that the transmission rate of COVID-19 was higher under the Fixed Movement Route (FMR) than under the Unfixed Movement Route (UMR) in 10 different sporting spaces per capita (1, 2, 3, 4, 5, 6, 7, 8, 9, and 10 m^2^) (*p* = 0.000). For both FMR and UMR, the larger the sporting space per capita, the lower the virus transmission rate. Additionally, when the sporting space per capita increases from 4 m^2^ to 5 m^2^, the virus transmission rate decreases most significantly (*p* = 0.000). In the FMR model with a per capita sporting space of 5 m^2^, minimizing gathering (no more than three people) could significantly slow down the transmission rate of the COVID-19 virus (*p* < 0.05). This study concluded that: (1) The UMR model is suggested in training facilities or playing grounds; (2) The sporting space should be non-overcrowding, and it is recommended that the sporting space per capita in the sporting grounds should not be less than 5 m^2^; (3) It is important to maintain safe social distancing and minimize gathering (no more than three people) when exercising.

## 1. Introduction

Coronavirus Disease 2019 (COVID-19), a global pandemic caused by severe acute respiratory syndrome coronavirus 2 (SARS-CoV-2), poses serious global public health concerns [1]. According to statistics published by the World Health Organization [2], globally, as of 9 July 2021, there have been more than 185,291,000 confirmed cases of COVID-19 and more than 4,010,000 deaths, affecting more than 210 countries and territories. COVID-19 spreads between people mainly through contact of the nose or ocular cells with the virus or indirectly through the inhalation of infectious respiratory aerosols [3,4]. SARS-CoV-2 is characterized by both high transmissibility and covert infections [5]. The general reproduction number (R0) of the virus was estimated to be 2.2~2.7 [6,7], but could go as high as 5.7 in some settings [8]. COVID-19 presents with headache, loss of the sense of smell and taste, nasal congestion and rhinorrhea, cough, muscle pain, sore throat, fever and breathing difficulties [9,10]. However, emerging evidence has revealed that some patients infected with SARS-CoV-2 are asymptomatic in the early stages, but the pre-symptomatic individual can still transmit the virus to others. The pre-symptomatic but infectious nature of several COVID-19 cases has frustrated measures to contain the epidemic [11,12].

Several measures such as self-isolation or quarantine, travel restrictions and the closure of public places (parks, gyms, etc.) have been proposed to contain the spread of SARS-CoV-2 [13,14]. Although these public health policies have slowed the spread of the virus to a greater extent, they have weighed down daily physical activeness and increased sedentary behavior such as watching TV, using mobile electronic devices and playing video games [15]. Given that these behaviors have a significant negative effect on human health (immune system, cardiovascular system, musculoskeletal system) [16], as we edge towards normalcy, people are being encouraged to participate in sporting activities to improve their physical health.

However, reports on transmission modes and prevention strategies of SARS-CoV-2 in sporting facilities remain unclear. Agent-based Modeling has been applied in numerous studies on the spread of other disease agents that have resulted in epidemics. For instance, Kim et al. [17] developed a stochastic agent-based framework and incorporated individual heterogeneity into the epidemic model. This work helped to develop effective intervention strategies and prevent plans for future emerging infectious diseases. On the other hand, Perez et al. [18] combined ABM and a geographic information system (GIS) to simulate the spread of a communicable disease in an urban environment. Their findings revealed that minimizing the dynamic interaction in closed spaces effectively reduces the spread of such diseases. In addition, D’Orazio et al. [19] utilized ABM to devise effective individual (facial masks) and collective (social distancing, travel restriction and lockdown) measures that helped slow down the spread of SARS-CoV-2. Ying et al. [20] presented a model for modeling virus transmission (SARS-CoV-2) based on ABM to reduce virus transmission in supermarkets. This research suggested restricting the maximum number of customers in a store or implementing a one-way aisle system. The above findings demonstrate the role of ABM in generating novel strategies for containing pandemics and epidemics. Consequently, in this research, we simulated the spread of COVID-19 in sporting facilities using the Agent-based Modeling (ABM) method, and analyzed the transmission rate in different sporting models, sporting spaces per capita and situations of gathering. Our findings may contribute to better understanding the SARS-CoV-2 transmission dynamics and propose measures necessary in the prevention of localized outbreaks.

## 2. Methods

### 2.1. Agent-Based Modeling

We used ABM to simulate the interaction between “agents” to evaluate the effects of their behavior on the entire system [21]. In ABM, a system is defined as a collection of autonomous decision-making entities called agents. Agents can constitute an autonomous and/or goal-directed entity, including but not limited to cells, people, organizations and/or entire synthetic populations [17,22,23]. Each agent assesses a situation and makes individual decisions based on set rules and executes various behaviors appropriate for the system they represent [24].

### 2.2. NetLogo Simulation

#### 2.2.1. Simulated Environment

The ABM for the transmission of COVID-19 was constructed using NetLogo software version 6.0.1 (Center for Connected Learning and Computer-Based Modeling, Northwestern University, Evanston, IL, USA), an open platform that models the interaction between natural and social phenomena [22,25]. In this study, the simulated environment mimicked sports facilities with dimensions of 126 × 51 patches (assumed that the patch size: 0.4 m × 0.4 m). The patches were divided into 10 equal areas. In addition, we estimated the size of an agent to be 0.4 m × 0.4 m, which meant that a patch could only be occupied by one non-overlapping agent.

#### 2.2.2. Attributes of the Agent

In this study, agents represented people with three different attributes: Susceptible (*S*), Exposed (*E*), and Infected (*I*) (Table 1). Notably, attributes varied during the interaction. The assumptions were: (1) The number of *I* in the sporting facility is fixed, and there were no new *I* during the simulation period; (2) *I* closely interacts with *S* during the training sessions (close interaction means that the distance between agents is less than 2 patches) and the infection (*S* infected into *E*) rate of positive contacts is 4.11% [26]; (3) *E* do not cause secondary infection; (4) The total number of people (*N*) in the sports facilities remained constant in the entire simulation period (*S* + *E* + *I* = *N*).

#### 2.2.3. Agent Movement Rules

**Figure 1:**
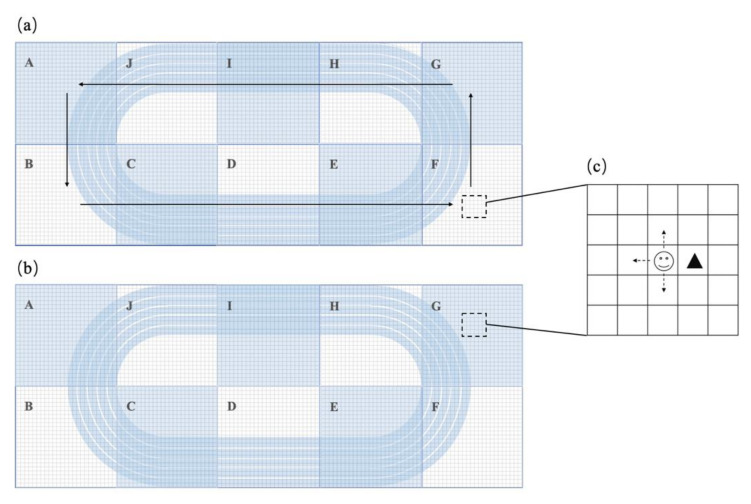
The movement route and direction of agents. (**a**) In the FMR model, the agents move counterclockwise through 10 different areas; (**b**) In the UMR model, the agents move randomly in the whole simulated space; (**c**) When obstructed by other agents or obstacles, the agent will randomly stop or change direction in order to bypass the agents or obstacles.

**Figure 2:**
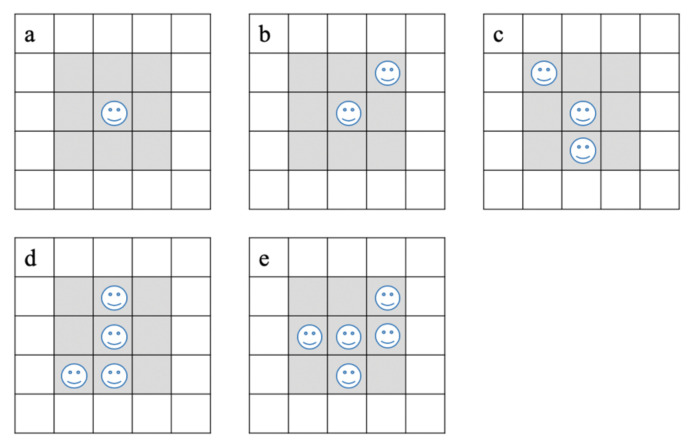
The simulated aggregation of different numbers of agents per basic area (grey area). (**a**) Only 1 agent per basic area; (**b**) No more than 2 agents per basic area; (**c**) No more than 3 agents per basic area; (**d**) No more than 4 agents per basic area; (**e**) No more than 5 agents per basic area.

The movement of the agent was guided by several rules; in the simulated environment, the agent moves within a random speed, and when obstructed by other agents or obstacles, the agent will randomly stop or change direction in order to bypass the agents or obstacles (Figure 1c). Furthermore, all agents move through two different models: the Fixed Movement Route (FMR) and the Unfixed Movement Route (UMR) models. In the FMR model, the agents move counterclockwise through 10 different areas (Figure 1a), whereas in the UMR model, the agents move randomly in the whole simulated space (Figure 1b). Aggregation was defined as the patch (1 patch) around the agent occupied by other agents. The 5 different situated aggregations of the agents are shown in Figure 2.

#### 2.2.4. Established Simulation

In this study, the spread of COVID-19 in different sporting models, sporting spaces per capita, and varied crowding was simulated using NetLogo software version 6.0.1. We used NetLogo to obtain the Running Time (*T*) when the number of *S* is same as that of *E*, and calculated the average time required for each *S* to be infected (Ticks value).

Ticks value = Running Time (*T*)/Number (*S*)

The virus transmission rate was represented by the Ticks value, and the larger the Ticks value, the slower the transmission rate. The patches represented the sporting space, and the size of the sporting space in the simulated environment was 50 m × 20 m (126 × 51 patches). The agents represented the specified number of participants, and the number of participants in the simulation parameters was 10 samples (1000, 500, 333, 250, 200, 166, 142, 125, 111, and 100 people, respectively), which corresponded to 10 different sports spaces per capita (1, 2, 3, 4, 5, 6, 7, 8, 9, and 10 m^2^). In addition, the movement routes of the agent (FMR and UMR) represented the different sports models. Under two interaction models (FMR and UMR), we analyzed the transmission rate of the virus in different per capita sporting spaces (1, 2, 3, 4, 5, 6, 7, 8, 9, and 10 m^2^) and varied degrees of gathering (no more than 1, 2, 3, 4, 5 people per basic area). Due to the random movement of the agent, we performed simulations under 30 different states, totaling 900 simulations. The simulation data were exported from NetLogo software version 6.0.1 into SPSS software version 22.0 (IBM Corporation, Armonk, NY, USA) for analysis.

### 2.3. Statistical Analysis

Data were analyzed using SPSS software version 22.0 (IBM Corporation, Armonk, NY, USA). Continuous data were expressed as mean ± standard deviation. The Paired Samples Test was used to analyze the difference in Ticks value under different sporting models and situations of gathering. The difference in Ticks value between sporting spaces per capita was analyzed using one-way ANOVA. *p* < 0.05 was considered statistically significant.

## 3. Results

### 3.1. Differences in Virus Transmission Rate between FMR and UMR Models

The virus transmission rates under the FMR and UMR models were analyzed based on the Ticks value. As shown in Table 2, the average Ticks value of FMR (38.38 ± 23.02) was 12.09% of UMR (317.14 ± 187.27). Specifically, in 10 different sporting spaces per capita (1, 2, 3, 4, 5, 6, 7, 8, 9, and 10 m^2^), the average Ticks value of UMR was greater than that of FMR with a statistically significant difference (*p* = 0.000). These results indicated that the UMR virus transmission rates were lower than those of the FMR model.

### 3.2. Differences in Virus Transmission Rate between Varied Sporting Spaces per Capita in Each Model

We calculated the Ticks values of 10 different per capita sports spaces in the FMR and UMR models to estimate the difference in the virus transmission rate in different per capita sports field areas. The simulation results showed that the Ticks value continually increased from 1 m^2^ (FMR = 6.49/UMR = 53.15) to 10 m^2^ (FMR = 67.99/UMR = 574.42) (Table 2). Further analysis indicated that among the nine sets of data (1 m^2^ and 2 m^2^, 2 m^2^ and 3 m^2^, 3 m^2^ and 4 m^2^, 4 m^2^ and 5 m^2^, 5 m^2^ and 6 m^2^, 6 m^2^ and 7 m^2^, 7 m^2^ and 8 m^2^, 8 m^2^ and 9 m^2^, 9 m^2^ and 10 m^2^), the Ticks value between 4 m^2^ and 5 m^2^ differed the most (FMR = 10.95/UMR = 78.94). As shown in Table 3 (FMR model) and Table 4 (UMR model), we also found a very significant statistical difference between 4 m^2^ and 5 m^2^ (*p* = 0.000). The above analysis indicated that for both FMR and UMR, the larger the sporting space per capita, the lower the virus transmission rate. Additionally, the virus transmission rate decreases significantly when the sporting space per capita increases from 4 m^2^ to 5 m^2^.

### 3.3. Differences in Virus Transmission Rate under Varied Situations of Gathering

**Figure 3:**
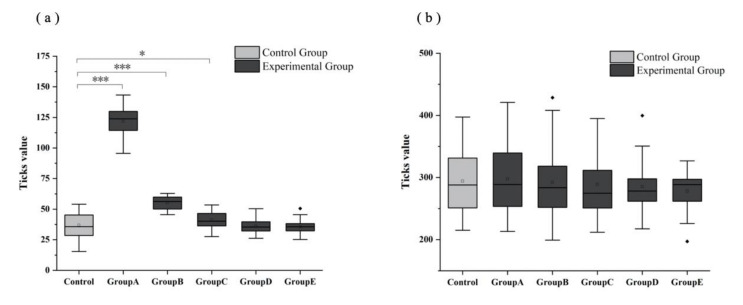
The transmission rate of SARS-CoV-2 under varied degrees of gathering in (**a**) FMR and (**b**) UMR models. Note: Control Group: No implementation to avoid gathering; Group A: No gatherings per basic area; Group B: No more than 2 people per basic area; Group C: No more than 3 people per basic area; Group D: No more than 4 people per basic area; Group E: No more than 5 people per basic area; * represents *p* < 0.05, *** represents *p* < 0.001.

Avoiding gatherings is one of the most effective measures of slowing down the transmission rate of SARS-CoV-2. Based on the above research results, the spread of the virus transmission rate has an obvious reduction from the per capita sporting space of 4 m^2^ to 5 m^2^. Therefore, we calculated the virus transmission rate under different degrees of gathering in the two different sporting models (FMR and UMR) with a sporting area per capita of 5 m^2^. Based on the Ticks value (Figure 3), we found a substantial difference in virus transmission rates in FMR under the per capita sporting area of 5 m^2^ between the Control Group (no implementation to avoid gathering) and Group A (no gathering per basic area) (*p* = 0.000)/Group B (no more than two people per basic area) (*p* = 0.000)/Group C (no more than three people per basic area) (*p* = 0.034). However, there was no difference in the virus transmission rate between Group D (no more than four people per basic area), Group E (no more than five people per basic area) and the controls (Figure 3a). Meanwhile, there was no difference in virus transmission in the UMR model at 5 m^2^ per capita sporting area and between other groups (Group A, Group B, Group C, Group D, Group E) and the controls (Figure 3b).

## 4. Discussion

We used FMR and UMR transmission models to simulate the spread of SARS-CoV-2 in different sports facilities under varied crowding. Based on the Ticks value, FMR allows for a higher transmission of SARS-CoV-2 than UMR. In the FMR model, individuals are more likely to be in the direct path of other people’s airflow, which increases the likelihood of infection. Therefore, long periods of running behind individuals should be avoided in sporting facilities.

The density of people in sporting facilities influences the spread of SARS-CoV-2. For instance, Jang et al. [27] found that large class sizes and small spaces increased the transmission rate of COVID-19 among fitness dancers. The moist, warm atmosphere in a sports facility coupled with turbulent airflow generated by intense physical exercise can cause more dense transmission of isolated droplets. Our simulation revealed that for both FMR and UMR, the larger the sporting space per capita, the lower the virus transmission rate. Additionally, the virus transmission rate decreases significantly when the sporting space per capita increases from 4 m^2^ to 5 m^2^. These findings suggest that the critical per capita sporting area for the transmission of SARS-CoV-2 may be 5 m^2^ in the FMR and UMR movement models. This disproportionate benefit may be because from 4 m^2^ to 5 m^2^ is a turning point for the spread of virus infection from static transmission to dynamic transmission. When the per capita sporting space is ≤4 m^2^, susceptible people may be directly located in the affected area of the virus, and it is more possible for susceptible people to become infected; when the per capita sporting space ≥5 m^2^, the infected person needs more time (patches) to spread the virus by contacting susceptible persons. The number of people in sporting facilities should always remain low (per capita sporting area of 5 m^2^) to reduce the risk of transmission.

SARS-CoV-2 is primarily transmitted between people through the inhalation of infectious respiratory droplets or the contact of susceptible cells with the virus, introduced from contaminated surfaces [4,28,29]. Therefore, decreasing gathering to maintain a safe social distance is essential in controlling the spread of the virus. Dominski et al. [30] suggested against larger classes of indoor exercises, and for outdoor events (walking, running, or cycling), appropriate social distances should be observed or athletes should practice a “side-by-side arrangement”. Overall, the FMR model with a per capita sports field area of 5 m^2^ significantly reduces the transmission risks of SARS-CoV-2 between individuals. Maintaining smaller crowds of no more than three people in sporting facilities within appropriate per capita spaces significantly slows down the transmission rate of SARS-CoV-2. Based on these findings, we propose sufficient social distancing and gatherings of no more than three people for closed door sporting facilities.

## 5. Limitation

This study has some limitations. First of all, we did not consider the different virus transmission rates between indoor and outdoor environments (the different types of sports facilities), which affect the spread of the virus [31,32]. We assumed that SARS-CoV-2 only spreads within a radius of two patches, and people only gather within one patch. We did not factor in other variables such as environment parameters (temperature, humidity, and airflow) [33]. Favorable environmental conditions (low temperature and high relative humidity levels) can enhance the transmission rate of SARS-CoV-2, though over shorter distances [34]. Secondly, this study did not consider testing the spread of SARS-CoV-2 in sports facilities before simulation to verify the feasibility of our results because there are no current data on the number of infections in sports facilities. In future studies, we will conduct more simulations on the different settings (the typology of the facility and the type of activity) and further verify the rationality of the model simulation results through field investigation and observation of the contact between participants.

## 6. Conclusions

In this study, we utilized the ABM method to analyze the transmission rate of COVID-19 in different sporting models, sporting spaces per capita, and situations of gathering. To the best of our knowledge, this is the first ABM study to contribute to understanding how COVID-19 transmits in sports facilities. The results showed that the transmission rates in the FMR models of each sporting space per capita (1, 2, 3, 4, 5, 6, 7, 8, 9, and 10 m^2^) were greater than those of the UMR model (*p* = 0.000). For both FMR and UMR, the larger the sporting space per capita, the lower the virus transmission rate. Additionally, when the sporting space per capita increases from 4 m^2^ to 5 m^2^, the virus transmission rate decreases most significantly (*p* = 0.000). In the FMR model with a per capita sporting space of 5 m^2^, minimizing gatherings (no more than three people) could significantly slow down the transmission rate of the COVID-19 virus (*p* < 0.05). In brief, based on the above ABM results, we found that UMR sporting models are superior to FMR models, and over-crowding and larger class sizes increase the transmission rate of SARS-CoV-2. Accordingly, we recommend the following: (1) the UMR model should be preferred over FMR in sporting facilities; (2) The per capita field area for sporting facilities should be greater than 5 m^2^; (3) Social distancing should be maintained at all times, with gatherings of no more than three people per enclosed area. The findings of this research provide a theoretical guide for minimizing the transmission of COVID-19 in sports facilities. The recommendations can also be applied in controlling the transmission of related viruses. The proposed recommendations can also be applied in controlling the spread of the virus in other crowded public places such as shopping malls, schools, and hospitals.

## Figures and Tables

**Table 1:** The different attributes (quantity, location, speed) of the agent.

Attribute	Quantity	Location	Speed	Attribute Update
Susceptible (*S*)	[0~999]	Randomly distributed	[0–1] patch/Tick	From *S* to *E*
Exposed (*E*)	[0~999]	Randomly distributed	[0–1] patch/Tick	None
Infected (*I*)	1	Randomly distributed	[0–1] patch/Tick	None

**Table 2:** The difference in virus transmission rates between varied models in 10 different sporting spaces.

Group (m^2^/Person)	Fixed Movement Route (Ticks)		Unfixed Movement Route (Ticks)		*p* Value
	Mean	SD	Mean	SD	
1	6.49	1.44	53.15	10.28	0.000
2	13.07	3.04	107.48	20.68	0.000
3	19.57	3.73	162.17	28.10	0.000
4	25.93	5.64	215.17	36.53	0.000
5	36.88	9.86	294.11	49.77	0.000
6	42.44	8.71	358.78	66.80	0.000
7	48.59	8.33	406.48	84.44	0.000
8	58.44	16.90	475.79	111.48	0.000
9	64.35	14.75	523.87	120.69	0.000
10	67.99	16.54	574.42	124.05	0.000
Average	38.38	23.02	317.14	187.27	0.000

**Table 3:** The difference in virus transmission rate between varied sporting spaces per capita in the FMR model.

Group (m^2^/Person)	1	2	3	4	5	6	7	8	9
2	0.015								
3	0.000	0.016							
4	0.000	0.000	0.018						
5	0.000	0.000	0.000	0.000					
6	0.000	0.000	0.000	0.000	0.039				
7	0.000	0.000	0.000	0.000	0.000	0.022			
8	0.000	0.000	0.000	0.000	0.000	0.000	0.000		
9	0.000	0.000	0.000	0.000	0.000	0.000	0.000	0.028	
10	0.000	0.000	0.000	0.000	0.000	0.000	0.000	0.000	0.176

**Table 4:** The difference in virus transmission rate between varied sporting spaces per capita in the UMR model.

Group (m^2^/Person)	1	2	3	4	5	6	7	8	9
2	0.007								
3	0.000	0.006							
4	0.000	0.000	0.008						
5	0.000	0.000	0.000	0.000					
6	0.000	0.000	0.000	0.000	0.001				
7	0.000	0.000	0.000	0.000	0.000	0.017			
8	0.000	0.000	0.000	0.000	0.000	0.000	0.001		
9	0.000	0.000	0.000	0.000	0.000	0.000	0.000	0.016	
10	0.000	0.000	0.000	0.000	0.000	0.000	0.000	0.000	0.011

## Data Availability

NetLogo code is available from the corresponding author upon reasonable request.
